# Available Relevant Study on Stress Analysis and Static Strength Prediction of Fiber Metal Laminates

**DOI:** 10.1155/2020/8874830

**Published:** 2020-11-27

**Authors:** Xiaochen Zhang, Weiying Meng, Jiancheng Guo, Yu Zhang

**Affiliations:** School of Mechanical Engineering, Shenyang Jianzhu University, No. 25 Hunnan Middle Road, Hunnan District, Shenyang, China 110168

## Abstract

Fiber metal laminates (FMLs) are a novel type of structural material that has been extensively applied in the aerospace field. These laminates are sandwich-type composite materials that comprise alternate metal and fiber-reinforced resin layers. Because of the structural characteristics of the material, it has high-impact resistance from the metal layer and increased fracture toughness and excellent fatigue and damage tolerance properties from the fiber layer. To further develop and apply this new composite material, it is essential to understand the research status on the stress analysis of each component in FMLs and the tensile strength properties of FMLs. Therefore, in this study, the current research status on the residual stress and applied stress of the component materials in FMLs and the tensile strength of the laminates is summarized. The relationship between the applied stress of each layer and the remote stress of laminates and the relationship between the tensile properties of laminates and the component material properties in laminates are clarified. Additionally, the theoretical basis and direction of development of the related models are analyzed and studied. Consequently, all of the above are aimed at laying a foundation for further investigations of the laminate theory and for the improvement of the theoretical research system.

## 1. Introduction

Fiber metal laminates (FMLs) constitute a joint scientific and technological achievement of Delft University, Fokker Aircraft Company, and the National Aerospace Laboratory of the Netherlands [1]. They are sandwich-type composites that comprise alternate metal and fiber-reinforced resin composite layers. They have high-impact resistance, increased fracture toughness, excellent fatigue, and damage tolerance properties and have attracted the attention of numerous enterprises and research institutes worldwide [2].

Different material properties can be obtained by changing the thickness, quantity, and type of the metal layer, the direction and system of fibers, and the thickness, quantity, and layup order of the fiber layer. While these composites perfectly combine the performance advantages of two different materials, FMLs also improve and remedy the individual deficiencies of the two materials. That is, they overcome the shortcomings of low-fatigue strength of aluminum alloys and the low ductility and impact strength, increased cost, and poor processability of fiber layers [3]. Compared to traditionally used aluminum alloy materials in aviation, FMLs can reduce weight by 25%–30% and increase fatigue life by a factor of 10–15 [4]. Their performance meets the requirements of the new generation of aviation equipment, although their manufacturing is complex and costly [5, 6]. Because of their light weight and high-damage tolerance, FMLs are currently used in aerospace structures instead of high-strength aluminum alloys [7], with examples including the fuselage, leading edge, and other parts of the Airbus A380 [8–10].

**Figure 1:**
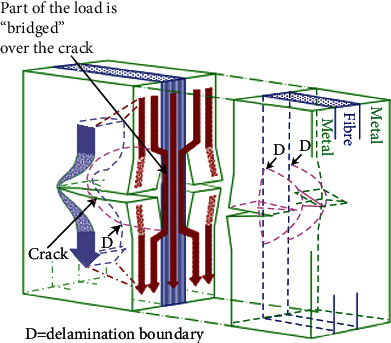
Stress path of cracked FMLs [17]. D: delamination boundary.

As shown in the schematic in Figure 1, the metal layers of FMLs can be accompanied by the fibers that resist crack growth [11, 12]. When cracks are generated and propagate in the metal layer, the fiber layer has a bridging effect that transfers a part of the load in the metal layer, and the stress of the metal layer and the stress intensity factor of the crack tip are reduced. It has also been reported that, in the process of fatigue, the stress in the metal layer plays a major role prior to the occurrence of the bridging mechanism, with the metal layer and bridging stresses working together upon the initiation of the bridging mechanism [13–16]. Therefore, the stress analysis of each layer in FMLs is critical when studying their fatigue properties. In addition, the study on the static strength properties of FMLs is based on stress analysis of each layer. In other words, the stress analysis of each layer in FMLs is a precondition and basis for studying their static and fatigue strengths.

Fiber metal laminates have unique structures and damage characteristics that yield the aforementioned advantages. However, FMLs also exhibit a number of characteristics that could, to a certain extent, restrict the application of laminates in the aerospace industry. Currently, a feature of Glass Reinforced Aluminum Laminates (GLARE) is their low Young's modulus compared to monolithic aluminum [8]. Young's modulus of the glass fiber layer is lower than that of the aluminum layer, and the combination of the aluminum and glass fiber layers inevitably results in a lower Young's modulus than that of a single aluminum alloy. Each layer with different material components has a different stiffness in the laminates. During a fatigue stress cycle, the layer with larger stiffness will generate more stress [12, 18]. The aluminum layer has a greater stiffness compared to the laminates, which will result in a higher stress level than the applied stress of the laminate. Another problem of FMLs is the existence of residual stresses. The FMLs are cured at an elevated temperature. After they cool down to room temperature, residual stresses will be generated in all layers of the cured laminate because of the differences in the thermal expansion coefficients. The residual stress in the aluminum layer of GLARE is tensile [12]. These two factors will result in a considerable increase in metal layer stress in the laminates under tensile loads. Therefore, it is essential to properly determine the stress of the metal layer in FMLs.

As an important structural material in the aerospace field, FMLs are primarily used as key fatigue parts. Although their fatigue performance constitutes a focus of material research studies, the static strength constitutes a basic mechanical property of materials, and its investigation is equally important. To further develop and apply these materials, it is necessary to evaluate the static strength properties and to clarify the relationship between the tensile properties of laminates and the properties of the component materials.

This study assesses the current status of knowledge on the stress levels of each layer in FMLS and summarizes the tensile laminate properties. The basic theory pertaining to established models is reviewed to establish (a) the relationship between the stress of each layer and the remote stress of laminates and (b) the relationship between the tensile properties of laminates and the component material properties. To promote further studies of the FMLs based on the analysis of the existing models, the direction of the anticipated theoretical development is clarified.

## 2. Stress Analysis

**Figure 2:**
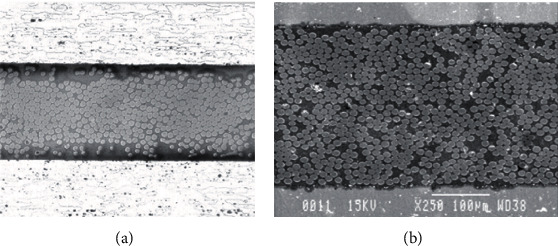
Fiber distribution in ARALL (a) and GLARE (b) [19].

**Figure 3:**
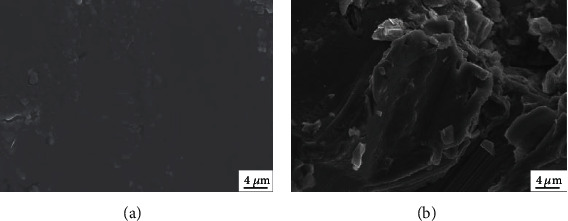
Surface morphology of metal layer in ARALL (a) and surface morphology after sandblast in GLARE (b).

So far, according to the types of fibers and metals, fiber metal laminates have mainly undergone four upgrades. They are Aramid Reinforced Aluminum Laminates (ARALL), Glass Reinforced Aluminum Laminates (GLARE), Carbon Reinforced Aluminum Laminate (CARE), and Titanium/Graphite Hybrid Laminates (TIGR). Among the four generations of fiber metal laminates, ARALL laminates and GLARE laminates have been successfully commercialized. There are some differences in structure between the two types of laminates. For ARALL laminates, there is a certain thickness of adhesive layer between the aramid fiber and aluminum alloy, but there is no adhesive layer between the glass fiber and aluminum alloy for the GLARE laminate, as shown in Figure 2. There are also some differences in metal surface treatment methods between the two kinds of laminates. For ARALL laminates, the metal surface of the bonding surface is not treated, while for GLARE laminates, the metal surface of the bonding surface is treated by sandblast, phosphoric acid anodization, and other methods, as shown in Figure 3.

### 2.1. Residual Stress Analysis

**Figure 4:**
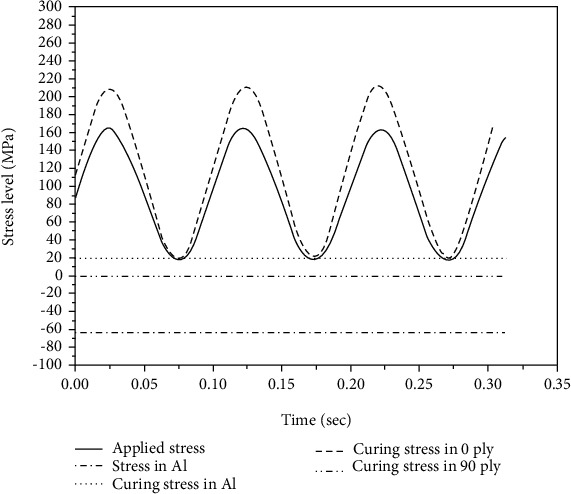
The stress level in Al layer and curing stress in each layer.

**Figure 5:**
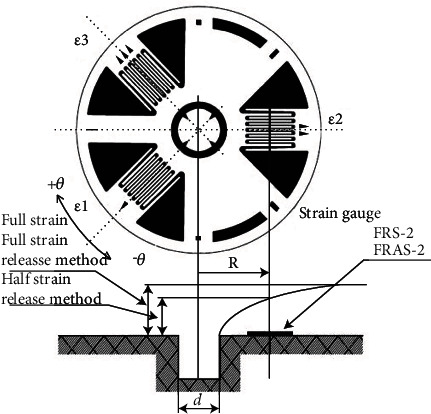
Schematic diagram of strain gauge test.

The preparation of fiber-reinforced metal laminates requires the bonding of each component material through curing. The curing of FMLs requires increased temperatures. When it is cooled to room temperature, residual stresses will be generated in each layer of the cured laminates because of the different thermal expansion coefficients of each material. Given that the metal layer produces residual tensile stress, the actual stress imparted to the metal layer will be further increased because of the tensile load, as shown in Figure 4. Therefore, for FMLs, the study of residual stress is critical. In addition, the residual stress of the metal was usually measured by strain gauge test, and the testing principle is shown in Figure 5.

#### 2.1.1. Analytic Method for Solving Residual Stresses

Based on the self-balancing principle of the residual stress of composites after curing, Oken and June [20] assumed that the response of each component material in the laminate exhibits elastic properties during the cooling process. Furthermore, according to the different thermal expansion coefficients of each component in the laminates, a commonly accepted formula for the calculation of residual stress of metal layers in FMLs is proposed, as follows:

(1) $$\begin{array}{c} \sigma_{\text{r},\text{AL}}=E_{\text{AL}}{\left(1+\frac{E_{\text{AL}}t_{\text{AL}}}{E_{\text{fm}}t_{\text{fm}}}\right)}^{-1}\left[\left(\alpha_{\text{fm}}-\alpha_{\text{AL}}\right)\left(T_{\text{T}}-T_{\text{C}}\right)\right], \end{array}$$

where *E*_fm_ and *t*_fm_ are the elastic modulus and thickness of the fiber resin layer in the laminates, respectively; *E*_AL_ and *t*_AL_ are the elastic modulus and thickness of the metal layer in the laminates, respectively; *T*_T_ and *T*_C_ are the test and curing temperatures, respectively; and *α*_AL_*α*_fm_ are the expansion coefficients of the metal and fiber resin layers, respectively.

Based on the classical laminate theory and the constitutive relationship of materials, the expression for the curing residual stress ${\bar{\sigma }}_{c,p}$ for each layer in FMLs was deduced by Homan [12] based on the consideration of the effect of thermal expansion coefficients of different materials on the internal stress of laminates during the curing process. The expression is as follows:

(2) $$\begin{array}{c} {\bar{\sigma }}_{c,p}={\left(S_{\varphi }\right)}_{p}\left({\bar{\varepsilon }}_{c}-\Delta T{\bar{\alpha }}_{p}\right), \end{array}$$

where (*S*_*φ*_)_*p*_ is the stiffness matrix of each layer material at an angle *φ*, ${\bar{\varepsilon }}_{c}$ is the curing strain of the material, *ΔT* is the temperature difference before and after curing, and ${\bar{\alpha }}_{p}$ is the thermal expansion coefficient of each layer.

**Figure 6:**
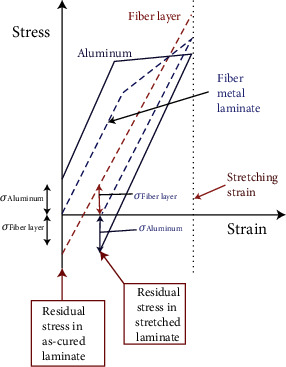
Illustration of poststretching process with stress and strain curves [17].

Khan et al. [17] further extended this theory based on the combination of the classical laminate and thermal expansion theories and proposed a new method to calculate the residual stress of each layer in the FML before and after the stress redistribution. The stress and strain curves in poststretching process are shown in Figure 6, and the expression for the metal layer stress is as follows:

(3) $$\begin{array}{c} {\left[\begin{array}{c} \alpha_{1}\\ \alpha_{2}\\ \tau_{12} \end{array}\right]}_{\text{al}}={\left[\begin{array}{ccc} Q_{11} & Q_{12} & 0\\ Q_{12} & Q_{22} & 0\\ 0 & 0 & Q_{66} \end{array}\right]}_{\text{al}}\eta_{\text{al}}, \end{array}$$

(4) $$\begin{array}{c} \eta_{\text{al}}={\left[\begin{array}{c} \varepsilon_{1,\text{pl}}\\ \varepsilon_{2,\text{pl}}\\ 0 \end{array}\right]}_{\text{lam}}+{\left[\begin{array}{ccc} A_{11} & A_{12} & 0\\ A_{12} & A_{22} & 0\\ 0 & 0 & A_{66} \end{array}\right]}^{-1}N-\left[\begin{array}{c} 1\\ 1\\ 0 \end{array}\right]\alpha_{\text{al}}\Delta T-{\left[\begin{array}{c} \varepsilon_{1,\text{pl}}\\ \varepsilon_{2,\text{pl}}\\ 0 \end{array}\right]}_{\text{al}}, \end{array}$$

where *N* = *N*_*i*_ + *N*_*i*_^T^, *N*_*i*_ is defined as the external load acting on laminates, *N*_*i*_^T^ is defined as the force owing to thermal expansion, **A**_ij_ is defined as the extensional stiffness of the laminate, *α*_al_ is defined as the coefficient of thermal expansion for Al, and Δ*T* is defined as the temperature difference before and after curing. Before stretching, *ε*_1,pl_ and *ε*_2,pl_ are both equal to zero, and *N*_*i*_ = 0. After stretching, *ε*_1,pl_ is known, *ε*_2,pl_ can be solved using the corresponding expressions, and N_i_ = 0.

Hu et al. [21, 22] adjusted the residual stress of Aramid Reinforced Aluminum Lamina (ARALL) 3/2 laminates using prestress and prestrain methods. A prestress method using springs was used to prestretch the glass fibers before laminate curing, and strain gauges were used to detect the residual stresses of the laminates at each stage during the formation process. Finally, the relationship between the residual stress *σ*_1_, prestress, and the curing temperature are analyzed, and the relevant expression is as follows:

(5) $$\begin{array}{c} \frac{\sigma_{1}}{E_{1}}=-\frac{\varepsilon_{0}}{1+E_{1}t_{1}/E_{2}t_{2}}+\frac{1}{1+E_{1}t_{1}/E_{2}t_{2}}\left(\alpha_{1}-\alpha_{2}\right)\left(T_{0}-T\right), \end{array}$$

where *t*, *E*, and *α* are the thickness, elasticity modulus, and coefficient of thermal expansion of the component materials, respectively; *ε*_0_ is the strain of the fiber after it was prestressed; *T* is the current temperature; *T*_0_ is the curing temperature; and the indices 1 and 2 denote the aluminum alloy and fiber layer, respectively. In addition, the prestrain method is used to stretch the cured laminate to produce a certain amount of plastic deformation (typically in the range of 0.4%–0.7%). Accordingly, the relationship between the residual stress *σ*_1_ and the applied prestrain is analyzed and is found to be linear. The relevant expression is as follows:

(6) $$\begin{array}{c} \sigma_{1}=\frac{D_{11}-B_{11}^{2}/A_{11}}{t_{1}\left(1.5t_{1}+t_{2}\right)-\left(B_{11}/A_{11}\right)t_{1}}\times \frac{1}{\rho }, \end{array}$$

where *A*_11_, *B*_11_, and *D*_11_ are the elastic constants of the laminates, *ρ* is the curvature radius of laminates after corrosion, and *t*_1_ and *t*_2_ are the thicknesses of the metal and fiber layers, respectively.

An analytical model was proposed by Abouhamzeh et al. [23] to predict the residual stress generated during the curing of FMLs. The model was based on the classical laminate theory and on an additional term that was dependent on the curing shrinkage. Accordingly, this model (a) reflects the change of stiffness and the chemical shrinkage of the material during curing and (b) predicts the development of residual stresses during curing for both the free and constrained (molded) curing of the composite laminates.

In addition, Li et al. [24], Zhong et al. [25], amongst others, also studied the residual stresses formed during the curing of the FMLs and proposed a corresponding theoretical calculation model. Relevant experiments were conducted to test the residual stresses of FMLs, and the proposed model was validated.

#### 2.1.2. Other Methods Used to Solve Residual Stresses

In addition to the most commonly used analytical method, there are also experimental and finite element methods that can be used to determine the residual stress of each layer in FMLs. The traditional test methods include the strain gauge embedding method, laser Raman spectroscopy, X-ray, fiber Bragg grating, and the corrosion delayering method. These traditional experimental methods are not specifically discussed in this study. Compared to the two methods presented above, the development of the finite element model is still in its infancy.

**Figure 7:**
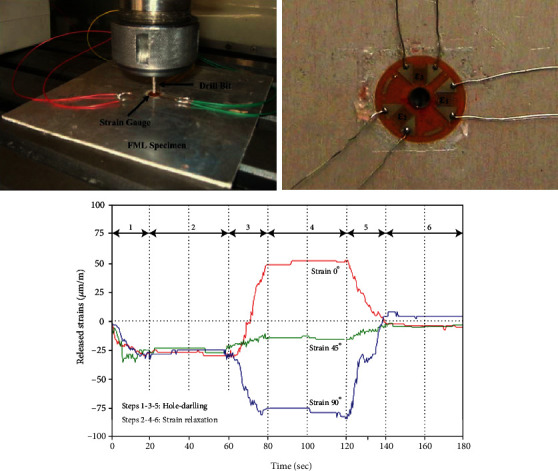
Measured strains in the IHD experiment [26].

Using an incremental hole-drilling technique combined with an integration method, Ghasemi and Mohammadi [26] measured experimentally nonuniform residual stresses in each ply of FMLs. At first, the calibration coefficient matrix was calculated using finite element simulation to relate the residual stresses and relieved strains. By performing the incremental hole-drilling experiment, released strains in the FML specimens were then measured. Subsequently, the residual stresses were obtained using the calibration coefficient matrix and the measured strains in each step of the incremental hole-drilling experiment. Finally, the experimental data from the incremental hole-drilling process were evaluated by the theoretical predictions of the classical laminate theory. The strain testing process in IHD experiment is shown in Figure 7.

**Figure 8:**
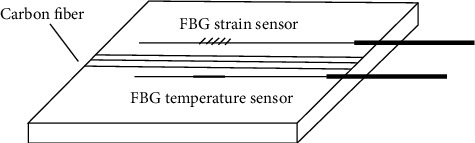
Laying method of FBG sensors [28].

A quasi-three-dimensional (3D) finite element method was used by Gu et al. [27] to calculate the interlaminar residual stress of FMLs. The computation results for residual stresses in ARALL laminates were generated in free or prestressed states at various levels of service temperatures, and the residual stresses in GLARE laminates in free states were presented. The optical fiber grating technology was used to monitor the strain and temperature of the whole curing process by Hu et al. [28], as shown in Figure 8, and the residual stress of the composite laminate with an aluminum sheet was simulated numerically using the ANSYS software. A finite difference method was employed to consider the strong coupling between the curing kinetics model and the thermal-chemical model during the simulation process for transient temperature fields. Chemical shrinkage was applied to composite materials as an initial strain for each time increment in the residual stress simulation.

The residual stresses of the composite laminates and the aluminum plates were successfully simulated on the basis of the referred technology. The residual stress of GLARE laminates was obtained using X-ray, analytical (Oken and June method), and corrosion delayering methods, which were proposed by Guo and Zheng [29]. The results indicated that the analytical and corrosion delayering methods were more accurate, and the X-ray method exhibited a significant difference and a large dispersion.

In the case of the FMLs, the analysis method for solving the residual stress has been studied more extensively. The study of residual stress in the free state is mature and has attained a high-prediction accuracy. However, when the residual stress is redistributed after stretching, the study of residual stress does not take into account the influence of plastic deformation of the component materials, and its prediction accuracy is not very satisfactory. The test method of residual stress has been developed more extensively, but the prediction accuracy has certain differences, among which the strain gauge embedding and the corrosion delayering methods have higher precisions. The finite element method of residual stress is still in its infancy and exhibits a certain degree of accuracy only for the corresponding situation. Therefore, related, in-depth research needs to be conducted.

### 2.2. Stress Analysis of Each Layer

The stress analysis of each layer in FMLs is critical for the study of the static and fatigue strengths of laminates. In the study of the static strength, the stress analysis of each layer is the basic premise for determining the failure of the material of each layer. In a fatigue strength study, the stress analysis of each layer is necessary for the prediction of the crack initiation and propagation lifetimes. Therefore, additional studies on the development of stress in each layer in the FML are essential.

For composite laminates, a variety of properties can be solved based on the classical laminate theory, i.e., the various material properties of laminates will be solved by modifying and extending the classical laminate theory [30–35]. This study found that the current solution for the stress of each layer of the FML is similar. The primary steps used to solve the stress in each layer of the FMLs are based on laminate theory and are as follows:

#### 2.2.1. Constitutive Relationships of Laminates and Their Components

Fiber metal laminates comprise parallel metal and nonmetal components. The constitutive relationship of all the components in the laminates is as follows:

(7) $$\begin{array}{c} \sigma_{\text{met}}=S_{\text{met}}\times \varepsilon_{\text{met}},\varepsilon_{\text{met}}=C_{\text{met}}\times \sigma_{\text{met}}, \end{array}$$

where *σ*_met_ is the stress tensor of the metal layer, *ε*_met_ is the strain tensor of the metal layer, *S*_met_ is the stiffness matrix of the metal material, and *C*_met_ is the compliance matrix of the metal material.

Accordingly, the stress-strain constitutive relation of laminates is as follows:

(8) $$\begin{array}{c} \sigma_{\text{lam}}=S_{\text{lam}}\times \varepsilon_{\text{lam}},\varepsilon_{\text{lam}}=C_{\text{lam}}\times \sigma_{\text{lam}}, \end{array}$$

where *σ*_lam_ is the stress tensor, *ε*_lam_ is the strain tensor, *S*_lam_ is the stiffness matrix, and *C*_lam_ is the compliance matrix of the laminates.

#### 2.2.2. Solving the Stiffness Matrix of Laminates

The stiffness matrix **Q** and compliance matrix **S** of the laminates are as follows, respectively:

(9) $$\begin{array}{c} Q=\overset{n}{\underset{k=1}{\sum }}\left({\left[\bar{c}\right]}_{k}\frac{t_{k}}{t_{\text{lam}}}\right), \end{array}$$

(10) $$\begin{array}{c} S=Q^{-1}, \end{array}$$

where *t*_*k*_ is the thickness of the *k*_th_ material layer and *t*_lam_ is the thickness of the laminates.

#### 2.2.3. Calculation of the Stress of Each Layer

When the laminate is subjected to an external stress *σ*_far_, the strain of the laminate is as follows:

(11) $$\begin{array}{c} \varepsilon_{\text{lam}}=C_{\text{lam}}\times \sigma_{\text{far}}. \end{array}$$

According to the laminate theory, the strain of the laminate is the same as that of the metal layer; therefore,

(12) $$\begin{array}{c} \varepsilon_{\text{lam}}=\varepsilon_{\text{met}}. \end{array}$$

Finally, the stress of the metal layer can be expressed as follows:

(13) $$\begin{array}{c} \sigma_{\text{met}}=S_{\text{met}}\times C_{\text{lam}}\times \sigma_{\text{far}}. \end{array}$$

#### 2.2.4. Research Trend of Stress Solving Methods for Each Layer in FMLs

**Figure 9:**
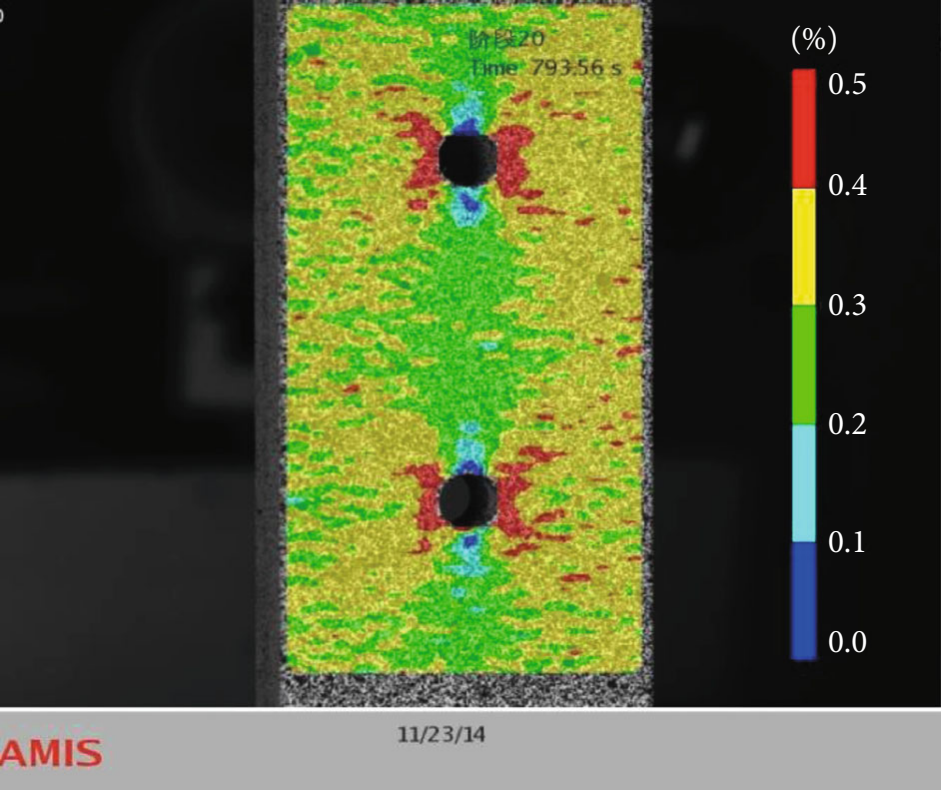
Strain diagram of specimen [38].

Currently, studies conducted on the stress solution method of each layer in FMLs are based on two aspects:

1. *Correction of the constitutive relationship of the component materials*: the constitutive relationship is corrected by considering the plasticity of the metal material. For example, Nowal [36] described a number of untypical failure modes, which have been observed during unloading in selected designs of multilayered structures. To clarify this behavior, based on which the delamination and buckling of the external aluminum layers occurs, when the external force is released, the classical lamination theory was applied to an elastic-plastic model of aluminum layers for the analysis of the stress distribution, while the total deformation theory proposed by Hencky and Ilyushin was applied to capture the influence of the plasticity of the metal on the mechanical performance of the hybrid structure. The elastic-plastic stress analysis and damage evolution of FMLs under internal pressure and thermal residual stress were studied by Zheng and Liu [37]. The elastic stress analysis of the composite laminates was performed based on the use of the classical laminate theory. In addition, the elastoplastic stress analysis of the liner layer was conducted by employing the power hardening theory and the Hencky equation in accordance with the plastic theory
2. *Correction of the stiffness matrix of the laminate material*: for example, based on the classical laminate theory, Meng et al. [38] modified the calculation method of elastic modulus of laminates by introducing the concept of effective stiffness of laminates; a digital optical strain gauge was used to verify the model, as shown in Figure 9; and a more accurate prediction of the metal layer stress in the FML was realized

Numerous studies have been conducted to solve the stress of each layer in FMLs before stress redistribution. Based on current and accumulated scientific knowledge, the stress of each layer can be solved based on the classical lamination theory in conjunction with the use of the modified constitutive relationship of component materials. However, current knowledge does not allow accurate solution of the stress of each layer based on the classical lamination theory in association with the modified stiffness matrix of laminates. The current research status on the quantification of stress of each layer in FMLs after stress redistribution is still at an initial stage. The solution method fails to take into account the degradation of the properties of each component material.

## 3. Tensile Properties

**Figure 10:**
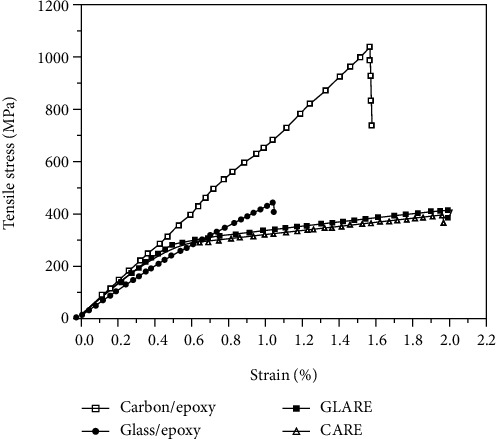
Tensile behavior of the laminates studied [39].

The tensile properties of FMLs are affected by their individual components. For example, the stress-strain behavior of FMLs clearly exhibits elastic responses within a 2.0% strain and is dependent on the properties of the prepreg and metal layers, as shown in Figure 10. In addition, its toughness and notch sensitivity depend primarily on the load-bearing capacity of the stress-strain response of the plastic region of the metal layer [39]. Similar to the bulk of fiber-reinforced composites, the properties of FMLs are directional because of the influence of fiber orientation, such as for ARALL and GLARE [40, 41]. It is noted that for FMLs, the interface bond between the prepreg and the metal layers plays a critical role in the transfer of stress for different materials in the laminates [2].

Because of its special structure, FMLs are different not only from isotropic metal materials but also from anisotropic fiber-reinforced composites, which make the study of their mechanical properties highly complex. The failure process of FMLs is complex under tensile loading. There are multifracture modes involved in the failure of GLARE laminates, such as matrix cracks, fiber-matrix debonding, fiber fractures, fiber/matrix interfacial shear failure, and interdelamination of laminates. Under longitudinal tensile loading, fiber pull-out and interface-matrix shear modes are common failure modes of fiber layers in FMLs [42]. In addition, the metal layer plays a role in preventing multiple global longitudinal splits. In the presence of transverse tensile loading, matrix failure and matrix-fiber interface debonding/fiber splitting are the primary fracture modes in the fiber-epoxy layer of FMLs. To better understand the formation and developmental damage in FMLs, and the effect of damage on residual strength, additional studies are required. The investigation of the tensile strength of FMLs involves analytical calculations, finite element analyses, and experimental methods, alone or in combination with each other. Currently, the analytical method has matured, and this study primarily introduces the research status of analytical methods.

To date, the techniques for the estimation of the tensile strength of FMLs by using analytical methods have been based on the metal volume fraction (MVF) and classical laminate theories.

### 3.1. Tensile Strength Calculation Based on Metal Volume Fraction Theory

MVF is a theory proposed by Vlot and Gunnink [2]. This theory can be used to predict the tensile modulus and strength of unidirectional FMLs. The MVF value, as defined by Vlot and Gunnink, can be calculated based on the following equation:

(14) $$\begin{array}{c} \text{MVF}=\frac{\sum_{i}^{n}t_{\text{al}}}{t_{\text{lam}}}, \end{array}$$

where *t*_lam_ is the thickness of the FML, *t*_al_ is the thickness of a single metal sheet, and *n* is the layer number of the metal sheet. On the basis of this definition, Vlot and Gunnink [2] also proposed prediction formulas for the tensile properties of GLARE laminates:

(15) $$\begin{array}{c} E_{\text{lam}}=\text{MVF}\times E_{\text{met}}+\left(1-\text{MVF}\right)\times E_{\text{FRP}}, \end{array}$$

(16) $$\begin{array}{c} \sigma_{0.2,\text{lam}}=\left[\text{MVF}+\left(1-\text{MVF}\right)\times \frac{E_{\text{FRP}}}{E_{\text{met}}}\right]\times \sigma_{0.2,\text{met}}, \end{array}$$

(17) $$\begin{array}{c} \sigma_{\text{t},\text{lam}}=\text{MVF}\times \sigma_{\text{t},\text{met}}+\left(1-\text{MVF}\right)\times \sigma_{\text{t},\text{FRP}}, \end{array}$$

where *σ*_t_ is the ultimate tensile strength, *σ*_0.2_ is the tensile yield strength, *E* is the tensile modulus, and subscripts lam, met, and FRP denote laminates, the metal layer, and fiber-reinforced composites, respectively.

Based on the characteristics of orthogonal laminates, Ma et al. [43] modified the MVF theory, which, as proposed byVlot and Gunnink, is only applicable to unidirectional FMLs, and further realized the prediction of the tensile properties of GLARE 3/2 laminates with 0/0° and GLARE 3/2 laminates with 0/90°. The corresponding expressions are as follows:

(18) $$\begin{array}{c} E_{\text{lam}}=\text{MVF}\times E_{\text{met}}+\alpha \times \left(1-\text{MVF}\right)\times E_{\text{FRP}}, \end{array}$$

(19) $$\begin{array}{c} \sigma_{0.2,\text{lam}}=\left[\text{MVF}+a\times \left(1-\text{MVF}\right)\times \frac{E_{\text{FRP}}}{E_{\text{met}}}\right]\times \sigma_{0.2,\text{met}}, \end{array}$$

(20) $$\begin{array}{c} \sigma_{\text{t},\text{lam}}=\text{MVF}\times \sigma_{\text{t},\text{met}}+a\times \left(1-\text{MVF}\right)\times \sigma_{\text{t},\text{FRP}}, \end{array}$$

where *α* is the volume fraction of fibers in the tension direction, *σ*_t_ is the ultimate tensile strength, *σ*_0.2_ is the tensile yield strength, *E* is the tensile modulus, and subscripts lam, met, and FRP denote laminates, the metal layer, and fiber-reinforced composites, respectively.

According to the characteristics of orthogonal GLARE laminates in combination with the mixing law of elastic moduli for composite materials, Wang et al. [44] modified the MVF theory based on the consideration of the effect of fibers on the performance in two in-plane directions and accurately predicted the elastic modulus, yield stress, and tensile strength of materials. The derived expressions are as follows, respectively:

(21) $$\begin{array}{c} E_{\text{lam}}=\text{MVF}\times E_{\text{met}}+a\times E_{\text{FRP}1}+b\times E_{\text{FRP}2}, \end{array}$$

(22) $$\begin{array}{c} \sigma_{\text{t},\text{lam}}=\left[\text{MVF}+a\times \frac{E_{\text{FRP}1}}{E_{\text{met}}}+b\times \frac{E_{\text{FRP}2}}{E_{\text{met}}}\right]\times \sigma_{\text{t},\text{met}}, \end{array}$$

(23) $$\begin{array}{c} \sigma_{0.2,\text{lam}}=\left[\text{MVF}+a\times \frac{E_{\text{FRP}1}}{E_{\text{met}}}+b\times \frac{E_{\text{FRP}2}}{E_{\text{met}}}\right]\times \sigma_{0.2,\text{met}}, \end{array}$$

where *σ*_0.2_ is the tensile yield strength, *σ*_t_ is the ultimate tensile strength, *E* is the tensile modulus, and *t* is the thickness of the material layer. In addition, subscripts lam and met represent laminates and the metal layer, respectively, and FRP1 and FRP2 represent the fiber layer in the directions of 0° and 90°, respectively. The parameters MVF, *a*, and *b* can be calculated as follows: MVF = ∑_1_^3^*t*_met_/*t*_lam_, *a* = ∑_1_^2^*t*_FRP1_/*t*_lam_, and *b* = ∑_1_^2^*t*_FRP2_/*t*_lam_.

The MVF method based on the mixing law can predict the tensile properties of FMLs. However, because of the elastic-plastic behavior of the metal layer, the elastic analysis cannot accurately predict the tensile response of FML materials. Therefore, the inelastic deformation behavior of FMLs must be considered after the alloy layer yields. To achieve a more accurate prediction of the stress-strain response and deformation behavior for FMLs, the analytical and finite element models consider that the evaluations of the plastic behavior after yielding and residual stress after curing are essential research directions.

### 3.2. Tensile Strength Calculation Based on Classical Laminate Theory

Taking into account the inadequacies of the MVF theory, researchers used the classical laminate theory to solve for tensile strength. The survey found that the primary steps needed for the prediction of the tensile strength of FMLs based on laminate theory are as follows:

1. Analyze the stress of each layer in FMLs based on the classical laminate theory. The current research status was explained above
2. Determine the damage state of the material using the failure criterion of the material. To analyze the failure situation of each layer in FMLs, four major strength theories (maximum tension, maximum linear strain, maximum principal shear stress, and maximum distortion-energy theories) are used for the metal layer, and the Tsai–Hill and Tsai–Wu theories are used in the prepreg layers
3. For each layer material that is judged to have failed, the degradation mode of material properties is analyzed based on the stiffness degradation criterion. When the material is considered to be damaged in the second step, the stiffness degradation of the material can be divided into complete and incomplete degradation. Currently, the incomplete degradation criterion is primarily used

There are two research directions used for the modification or expansion of the classical laminate theory to solve the tensile properties of FMLs:

1. Correction of the constitutive relationship of component materials in the laminate
2. Correction of the degenerate form of component materials in the laminate

The related studies are now discussed. Tensile properties of the Ti/APC-2 laminates with different fiber directions were predicted by Cortés and Cantwell [45]. To predict the tensile strength of laminates at different fiber directions, the classical laminate theory was modified by considering the effect of residual stress, and the failure criteria of Tsai–Hill, Tsai–Wu, and maximum stress were used. However, the model did not consider the effect of the metal plasticity stage. The modified classic laminate theory is as follows:

(24) $$\begin{array}{c} {\left\{\sigma \right\}}_{x,y}^{k}={\left\{\sigma^{\text{TH}}\right\}}_{x,y}^{k}+{\left\{\sigma^{\text{M}}\right\}}_{x,y}^{k}, \end{array}$$

(25) $$\begin{array}{c} {\left\{\sigma^{\text{TH}}\right\}}_{x,y}^{k}=Q_{x,y}^{k}\left({\left\{\varepsilon^{0}\right\}}_{x,y}+z{\left\{\kappa \right\}}_{x,y}-{\left\{\alpha \right\}}_{x,y}^{k}\Delta T\right), \end{array}$$

(26) $$\begin{array}{c} {\left\{\sigma^{\text{M}}\right\}}_{x,y}^{k}=Q_{x,y}^{k}\left({\left\{\varepsilon^{0}\right\}}_{x,y}+z{\left\{\kappa \right\}}_{x,y}\right), \end{array}$$

where {*σ*}_*x*,*y*_^*k*^ is the actual stress of the *k*_th_ layer in the *xy*-coordinate system of the laminate specimen, {*σ*^TH^}_*x*,*y*_^*k*^ is the residual thermal stress of the *k*_th_ layer in the *xy*-coordinate system of the laminate specimen, {*σ*^M^}_*x*,*y*_^*k*^ is the mechanical stresses of the *k*_th_ layer in the *xy*-coordinate system of the laminate specimen, *Q*_*x*,*y*_^*k*^ is the stiffness matrix of the *k*_th_ layer in the *xy*-coordinate system of the laminate specimen, {*ε*^0^}_*x*,*y*_ are the midplane strains of the laminate specimen in the *xy*-coordinate system, *z* is the distance from the midplane, and {*κ*}_*x*,*y*_ is the curvature in the *xy*-specimen coordinate system.

**Figure 11:**
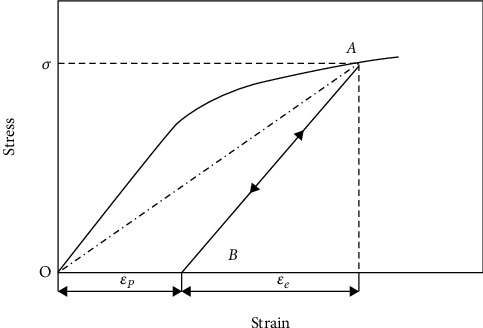
Elastic-plastic stress-strain curves for metal materials.

The tensile stress-strain behavior of GLARE laminates was studied by Iaccarino et al. [46]. To theoretically predict the laminate response based on the anisotropic characteristics of the metal layer, the classical lamination theory was modified to account for the inelastic behavior of aluminum, which was substituted by an “equivalent” material governed by a simple constitutive law. The maximum strain and Tsai–Hill criteria were used as the final failure conditions of aluminum and fiberglass, respectively. The equivalent constitutive relation of the metals can be expressed as follows:

(27) $$\begin{array}{c} v_{\text{eq}}=\frac{1}{2}\left[1-\frac{E_{\text{eq}}}{E_{\text{al}}}\left(1-2v_{\text{al}}\right)\right], \end{array}$$

where *E*_al_ is the elastic modulus of aluminum; *E*_eq_ is the equivalent modulus of aluminum, that is, the slope of the OA segment in Figure 11; and *v*_al_ is Poisson's ratio of aluminum.

Chen and Sun [47] described the elastic-plastic stress-strain relations in an ARALL laminate based on the use of classical laminate theory with proper elastic-plastic models for aluminum and modeled ARALL laminates as homogeneous orthotropic elastic-plastic solids based on the use of a three-parameter, plastic potential function. Comparison with experimental results indicated that the orthotropic plasticity model was approximately accurate up to a total strain of 1.2%, and the modified classical laminate theory was found to be capable of describing the stress-strain curves up to failure.

The nonlinear tensile responses and fracture behaviors of GLARE 4 and GLARE 5 laminates were studied by Wu and Yang [42] using inplane loading. An analytical model was proposed based on the modified classical lamination theory, which incorporated the elastoplastic behavior of the aluminum alloy to predict the stress-strain response and deformation behavior of GLARE laminates. The constitutive relationships of the laminate in the elastic and plastic states of the metal layer are as follows, respectively:

(28) $$\begin{array}{c} dN=\left\{n^{\text{Al}}h^{\text{Al}}{\left(S_{\text{e}}^{\text{Al}}\right)}^{-1}+n^{\text{c}}h^{\text{c}}Q^{\text{c}}\right\}d\varepsilon , \end{array}$$

(29) $$\begin{array}{c} dN=\left\{n^{\text{Al}}h^{\text{Al}}{\left(S_{\text{e}}^{\text{Al}}+S_{\text{p}}^{\text{Al}}\right)}^{-1}+n^{\text{c}}h^{\text{c}}Q^{\text{c}}\right\}d\varepsilon , \end{array}$$

where *dN* represents the inplane force increments per unit length; *dε* represents the midplane strain increments per unit length; *S*_e_^Al^ represents the flexibility tensor of the aluminum alloy layer under elastic conditions; *S*_p_^Al^ represents the flexibility tensor of the aluminum alloy layer under plastic conditions; *n*^Al^ and *n*^c^ are the numbers of metal and prepreg layers, respectively; *h*^Al^ and *h*^c^ are the thicknesses of the metal and prepreg layers, respectively; and *Q*^c^ is the stiffness matrix of the prepreg layer.

The classical laminate theory (CLT) was applied by Kawai et al. [48] to describe the off-axis inelastic behavior of GLARE 2 laminate. An incomplete stiffness degradation model was proposed, which took into account the transverse failure in glass fiber-reinforced plastic layers to cause an instantaneous degradation of transverse and shear elastic moduli based on CLT. Accordingly, the characteristic deformation behavior of GLARE 2 was accurately described. The incomplete stiffness degradation model is as follows:

(30) $$\begin{array}{c} E_{11}^{\text{d}}=E_{11},E_{22}^{\text{d}}=0,v_{12}^{\text{d}}=0,G_{12}^{\text{d}}=0, \end{array}$$

where *E*_11_^*d*^ degenerates into a new *E*_11_, and the other parameters tend to zero.

Cortés and Cantwell [45] reported the tensile properties of Ti/APC-2 laminates under different fiber directions and proposed that the stiffness degradation of the prepreg layer was related to the loading angle. When the loading angle was less than 15°, the degenerated stiffness is as follows:

(31) $$\begin{array}{c} E_{1}=0,E_{22}^{\text{d}}=E_{22},v_{12}=0,G_{12}=0. \end{array}$$

When the loading angle is greater than 15°, the degenerated stiffness is equal to

(32) $$\begin{array}{c} E_{11}^{\text{d}}=E_{11},E_{2}=0,v_{12}=0,G_{12}=0. \end{array}$$

**Figure 12:**
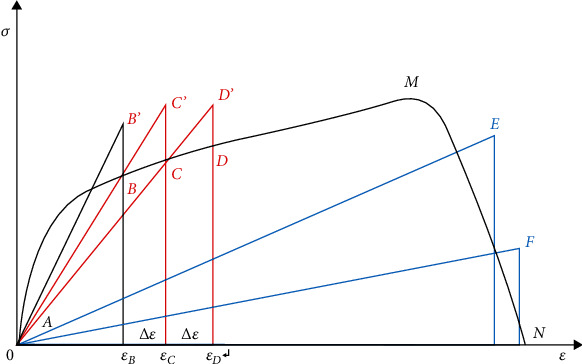
The stress-strain curve of composite under tensile loading.

**Figure 13:**
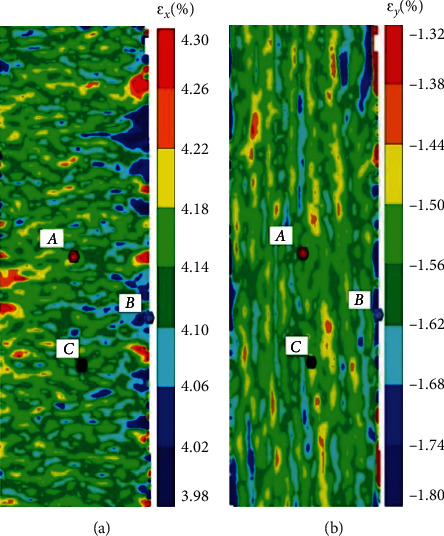
Strain distributing nephogram of GLARE2-3/2 laminates under uniaxial tensile loading [49]: (a) axial; (b) transverse.

A general method to describe the degradation of material properties was presented by Tong et al. [49], which is simple and suitable for theoretical model calculation. When the material is in the fracture stage, as shown in the MN section of the curve in Figure 12, the degradation process of material properties can be described in the theoretical model as long as the equation of the stress-strain curve is known. It is suggested that formula (33) should be used for fitting when the stress-strain curve of the material is close to the index change and formula (34) should be used when it is close to the linear change. In addition, the distributing nephogram of the strain for the laminate before fracture was obtained by experiment, as shown in Figure 13.

(33) $$\begin{array}{c} y=A_{1}{\text{e}}^{\left(-x/t_{1}\right)}+A_{2}{\text{e}}^{\left(-x/t_{2}\right)}+y_{0}, \end{array}$$

(34) $$\begin{array}{c} y=A_{1}x+B. \end{array}$$

A common analytical model that is independent of the configuration of the laminate was developed by Rao and Subba Rao [50] based on a hybrid degradation scheme. The model was proposed using the constant degradation factors based on the condition of the adjacent lamina. It is considered that the deterioration of the performance of each damaged layer is related to the state of its adjacent lamina. In other words, when two adjacent laminae are not damaged, the performance of the damaged lamina will degenerate to 70%. When only one adjacent lamina is damaged, it will degenerate to 50%; when both adjacent laminae are damaged, the performance of the damaged lamina will degenerate to approximately zero.

Currently, theoretical research on the tensile strength of FMLs is primarily based on the classical laminate theory. Because of the complexity of FMLs, there is no perfect theoretical system to analyze and calculate the tensile strength of FMLs. In addition, the effects of interlaminar properties on the tensile strength of FMLs are critical and cannot be ignored. Currently, the classical laminate theory fails to consider the effect of interlaminar properties, and it will be further studied by considering this effect.

## 4. Conclusions

In this study, the stress analysis and tensile strength of FMLs were introduced. The developments of the theoretical and experimental methods for the solution of residual stresses of laminates were expounded. The analytical models for the stress analysis of each layer in the laminates and their corresponding defects were analyzed. The two types of analytical models for tensile strength of laminates and their corresponding defects were studied and discussed.

In summary, for the analyses of the basic mechanical properties of FMLs, the residual stress and stress imposed on each layer in a free state have matured developmentally. However, previous studies on the corresponding performance after the stress redistribution did not consider the property degradation effects of the component materials. Accordingly, additional studies on this aspect are needed. In-depth studies have been conducted on the tensile properties of FMLs based on classical laminate theory; however, only limited studies have been conducted on the interfacial bonding strength of laminates. The interfacial bonding strength plays a critical role in static strength performance. Correspondingly, the influencing factors have not been identified. In addition, the compressive properties and its failure mechanism of FMLs have not been fully studied, and the related research work considering residual stress on the compressive properties needs further development.

## References

[1] Vlot A., Vogelesang L. B., de Vries T. J. (1999). Towards application of fibre metal laminates in large aircraft. *Aircraft Engineering and Aerospace Technology*.

[2] Vlot A., Gunnink J. W. (2001). *Fibre Metal Laminates: An Introduction*.

[3] Wang S. Y. (2012). *Preparation and Mechanical Properties of Fiber Metal Laminates [Dissertation]*.

[4] Tao J., Li H. G., Pan L., Hu Y. B. (2015). Review on research and development of fiber metal laminates. *Nanjing University of Aeronautics and Astronautics*.

[5] Jiang L. P. (2012). Research of glare laminate fatigue performance comprehensive evaluation. *Materials Research*.

[6] Vermeeren C. A. J. R., Beumler T., de Kanter J. L. C. G., van der Jagt O. C., Out B. C. L. (2003). Glare design aspects and philosophies. *Applied Composite Materials*.

[7] Guo Y. J., Wu X. R. (1998). Phenomenological model for predicting fatigue crack growth in fiber reinforced metal laminates. *Acta Aeronaut et Astronaut Sinica*.

[8] Chang P. Y., Yeh P. C., Yang J. M. (2008). Fatigue crack initiation in hybrid boron/glass/aluminum fiber metal laminates. *Materials Science and Engineering: A*.

[9] Frizzell R. M., Mccarthy C. T., Mccarthy M. A. (2008). An experimental investigation into the progression of damage in pin-loaded fibre metal laminates. *Composites Part B: Engineering*.

[10] Fu Y. M., Zhong J., Chen Y. (2014). Thermal postbuckling analysis of fiber–metal laminated plates including interfacial damage. *Composites Part B: Engineering*.

[11] Marissen R. (1988). *Fatigue Crack Growth in ARALL: A Hybrid Aluminum-Aramid Composite Material Crack Growth Mechanisms and Quantitative Predictions of the Crack Growth Rates, Report LR-574*.

[12] Homan J. J. (2006). Fatigue initiation in fibre metal laminates. *International Journal of Fatigue*.

[13] Guo Y. J. (1997). *Fatigue Damage and Life Prediction of Fiber Reinforced Metal Laminates [Dissertation]*.

[14] Kieboom O. (2000). *Fatigue Crack Initiation and Early Crack Growth in Glare at Different Temperatures [Dissertation]*.

[15] Sen I., Alderliesten R. C., Benedictus R. (2015). Design optimisation procedure for fibre metal laminates based on fatigue crack initiation. *Composite Structures*.

[16] Huang Y., Liu J. Z., Huang X., Zhang J. Z., Yue G. Q. (2015). Delamination and fatigue crack growth behavior in fiber metal laminates (Glare) under single overloads. *International Journal of Fatigue*.

[17] Khan S. U., Alderliesten R. C., Benedictus R. (2009). Post-stretching induced stress redistribution in fibre metal laminates for increased fatigue crack growth resistance. *Fibre Science and Technology*.

[18] Vasek A., Polak J., Kozak V. (1997). Fatigue crack initiation in fibre-metal laminate GLARE 2. *Materials Science and Engineering A*.

[19] Alderliesten R. C. (2007). On the available relevant approaches for fatigue crack propagation prediction in GLARE. *International Journal of Fatigue*.

[20] Oken S., June R. R. (1971). *Analytical and Experimental Investigation of Aircraft Metal Structures Reinforced with Filamentary Composites [Report]*.

[21] Hu H. J., Li H. Y., Zheng R. Q. (1993). Modifying residual stresses in ARALL laminates by prestressing. *Acta Mater Compos Sinica*.

[22] Hu H. J., Li H. Y., Zheng R. Q., Qiang W. (1995). Method of modifying residual stresses of aramid-aluminum laminates (ARALL) by prestrain. *Acta Mater Compos Sinica*.

[23] Abouhamzeh M., Sinke J., Jansen K. M. B., Benedictus R. (2015). Closed form expression for residual stresses and warpage during cure of composite laminates. *Composite Structures*.

[24] Li H. Y., Hu H. J., Zheng R. Q. (1994). Analysis and measurement on residual stress of aramid aluminum laminates. *Acta Aeronaut et Astronaut Sinica*.

[25] Zhong W. H., Gao Z. Q., Zhang Z. G., Yang H. C. (2000). Study of residual stress in super-hybrid composite Ti/CFRP. *New carbon Maternité*.

[26] Ghasemi A. R., Mohammadi M. M. (2016). Residual stress measurement of fiber metal laminates using incremental hole-drilling technique in consideration of the integral method. *International Journal of Mechanical Sciences*.

[27] Gu Z. F., Cui D. Y., Zhong W. H., Li H. Y., Hu H. J., Zheng R. Q. (1995). Analysis of residual stresses for fiber reinforced alumimium laminate. *Acta Mater Compos Sinica*.

[28] Hu Z. H., Wang G. R., He X. D., Liu W. B. (2007). The effect of metal mould on residual stress of composite laminated during cure process. *J Astronaut*.

[29] Guo Y. J., Zheng R. Q. (1998). The residual stresses in glass fiber reinforced aluminium laminates (GLARE). *Journal of Materials Engineering*.

[30] Brunbauer J., Pinter G. (2015). Fatigue life prediction of carbon fibre reinforced laminates by using cycle-dependent classical laminate theory. *Composites Part B: Engineering*.

[31] Park C. H., Baz A. (2001). Comparison between finite element formulations of active constrained layer damping using classical and layer-wise laminate theory. *Finite Elements in Analysis and Design*.

[32] Chaphalkar P., Kelkar A. D. (2001). Classical laminate theory model for twill weave fabric composites. *Composites. Part A, Applied Science and Manufacturing*.

[33] Wang Q., Liang L. L., Peng C. N. (2015). Analysis of the mechanical properties of TC4-6061 composite plate based on classical laminate theory. *Applied Mechanics and Materials*.

[34] Kale V. S., Chhapkhane N. K. (2013). Analysis of the response of a laminate to imposed forces using classical lamination theory and finite element technique. *Int J Eng Sci Tec*.

[35] Fukunag H., Vanderplaats G. N. (1991). Stiffness optimization of orthotropic laminated composites using lamination parameters. *AIAA Journal*.

[36] Nowal T. (2018). Elastic-plastic behavior and failure analysis of selected fiber metal laminates. *Composite Structures*.

[37] Zheng J. Y., Liu P. F. (2008). Elasto-plastic stress analysis and burst strength evaluation of Al-carbon fiber/epoxy composite cylindrical laminates. *Computational Materials Science*.

[38] Meng W. Y., Xie L. Y., Hu J. X., Lv X., Qin B., Wang B. W. (2018). Strain measurement and stress prediction methods of metal layer in fiber metal laminates. *J B Univ Aeronaut Astronaut*.

[39] Botelho E. C., Silva R. A., Pardini L. C., Rezende M. C. (2006). A review on the development and properties of continuous fiber/epoxy/aluminum hybrid composites for aircraft structures. *Materials Research*.

[40] Gunnink J. W., Vogelesang L. B. Aerospace ARALL-the advancement in aircraft materials.

[41] Gunnink J. W., Vogelesang L. B. Aerospace ARALL: a challenger for aircraft designer.

[42] Wu G. C., Yang J. M. (2005). Analytical modelling and numerical simulation of the nonlinear deformation of hybrid fibre–metal laminates. *Modelling Simul Mater Sci Eng*.

[43] Ma H. Y., Mackay D., Chi Lee S., Ying Shiu W. (2006). Aliphatic and cyclic hydrocarbons. *Journal of Materials Engineering*.

[44] Wang Y. J., Wang B., Zhang L., Ma H. Y. (2015). Tensile properties of glass fiber reinforced aluminum orthorhombic laminate. *Journal of Materials Engineering*.

[45] Cortés P., Cantwell W. J. (2006). The prediction of tensile failure in titanium-based thermoplastic fibre-metal laminates. *Composites Science and Technology*.

[46] Iaccarino P., Langella A., Caprino G. (2007). A simplified model to predict the tensile and shear stress–strain behaviour of fibreglass/aluminium laminates. *Composites Science and Technology*.

[47] Chen J. L., Sun C. T. (1989). Modeling of orthotropic elastic-plastic properties of ARALL laminates. *Composites Science and Technology*.

[48] Kawai M., Morishita M., Tomura S., Takumida K. (1998). Inelastic behavior and strength of fiber-metal hybrid composite: glare. *International Journal of Mechanical Sciences*.

[49] Tong A. S., Xie L. Y., Bai E. J., Bai X., Zhang S. J., Wang B. W. (2017). Test and prediction model of statics property of fiber metal laminates. *Acta Aeronautica Et Astronautica Sinica*.

[50] Rao P. M., Subba Rao V. (2010). Estimating the failure strength of fiber metal laminates by using a hybrid degradation model. *Journal of Reinforced Plastics and Composites*.

